# Supplementary material for: Chemical unfolding of protein domains induces shape change in programmed protein hydrogels
Source: Nat Commun. 2019 Nov 29;10:5439. doi: 10.1038/s41467-019-13312-0 (PMC6884551; doi:10.1038/s41467-019-13312-0)
Supplement: Supplementary file 2 — Supporting Information [file 41467_2019_13312_MOESM2_ESM.pdf]

Supplementary Information

Chemical unfolding of protein domains induces shape change in programmed protein hydrogels

Luai R. Khoury, Ionel Popa

Department of Physics, University of Wisconsin-Milwaukee, 3135 North Maryland Ave., Milwaukee, Wisconsin 53211, United States

CONTENT

Supplementary Methods. Supplementary Figures 1-5. Supplementary Movie Caption.

Supplementary Methods

Effect of polymer type on the pore area distribution on treated BSA-based hydrogels. Scanning electron microscope (SEM) characterization was used to study the effect of the polymer on the pore-area size distribution of different hydrogel samples. First, we prepared native BSA-based hydrogel as a control, and three different BSA-based hydrogel samples, each treated with 1 mM of polyethyleneimine (PEI), poly-(L)-lysine (PLL), and poly ethylene glycol (PEG) for 30 min, as reported in the Methods section. Then, the samples were frozen in liquid nitrogen prior to lyophilizing for 24 hours. The dried samples were broken by forceps to expose the cross-section area and mounted on aluminum stubs using carbon double-side tape. Then, the samples were sputter-coated with iridium (3 nm) prior to imaging with SEM (HITACHI S-4800) with 5 keV acceleration voltage. The hydrogel pore-size and wall thickness were characterized using ImageJ software (NIH, USA). Supplementary Figure 1 shows that BSA-based hydrogel sample treated with PEI and PLL displayed a significant decrease in the average pore-area size (PEI: 421 ± 141 μm^2, PLL: 502 ± 247 μm^2) compared with the native BSA-based hydrogel 1126 ± 636 μm^2. Additionally, the treatment of BSA hydrogel with PEG polymer (1121± 700 μm^2) showed no significant change in the average pore-size area compared with native BSA-based hydrogel. However, a significant change was noticed between the PEI and PLL treated hydrogels compared to native BSA hydrogels.

Supplementary Figures

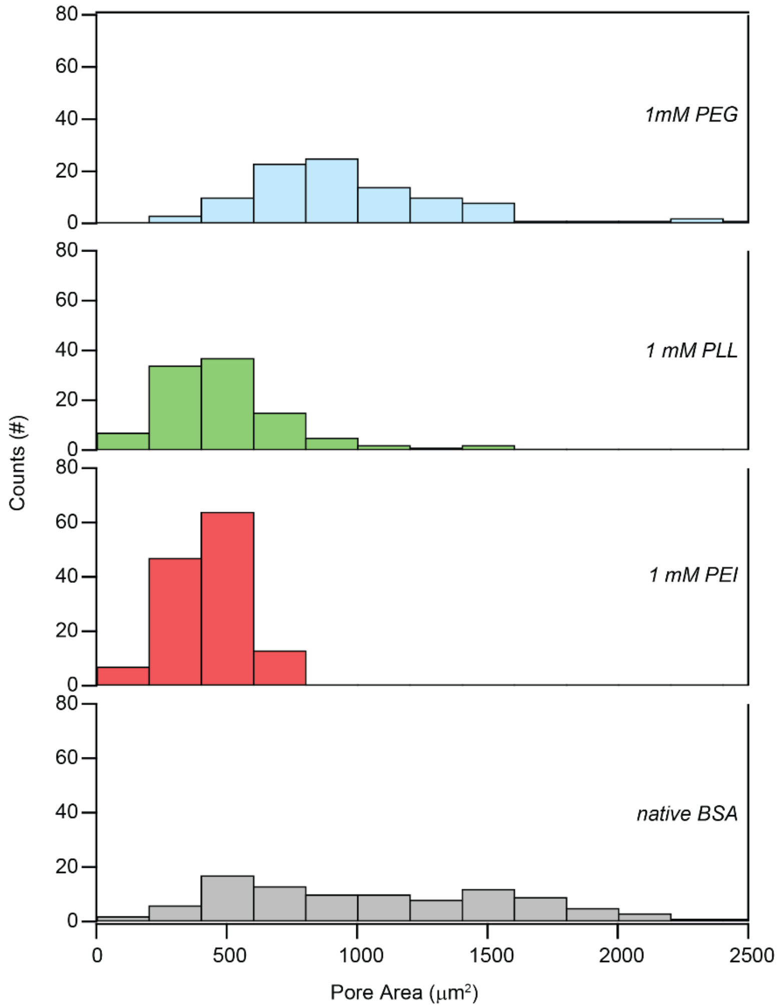

Supplementary Figure 1. Change in pore area with type of polyelectrolyte. Pore-Area distribution histograms of native-BSA (black) and after incubation with 1 mM PEG (blue), PLL (green) and PEI (red). The pore areas were measured from SEM images using ImageJ software.

Effect of PEI concentration on the wall thickness distribution on treated BSA-based hydrogels

Scanning electron microscope (SEM) characterization was used to examine the effect of the polymer on the wall thickness of different BSA-based hydrogel samples treated with various PEI concentrations. First, we prepared native BSA-based hydrogel as a control sample, and different BSA-based hydrogel samples treated with PEI concentrations ranging from 0.25 to 2 mM, as previously reported. Then, all samples were prepared and characterized using SEM as mentioned in the previous section. The samples wall thickness was characterized using ImageJ software (NIH, USA). Supplementary Figure 2 shows that wall thickness of BSA-based hydrogel samples increases with PEI concentration.

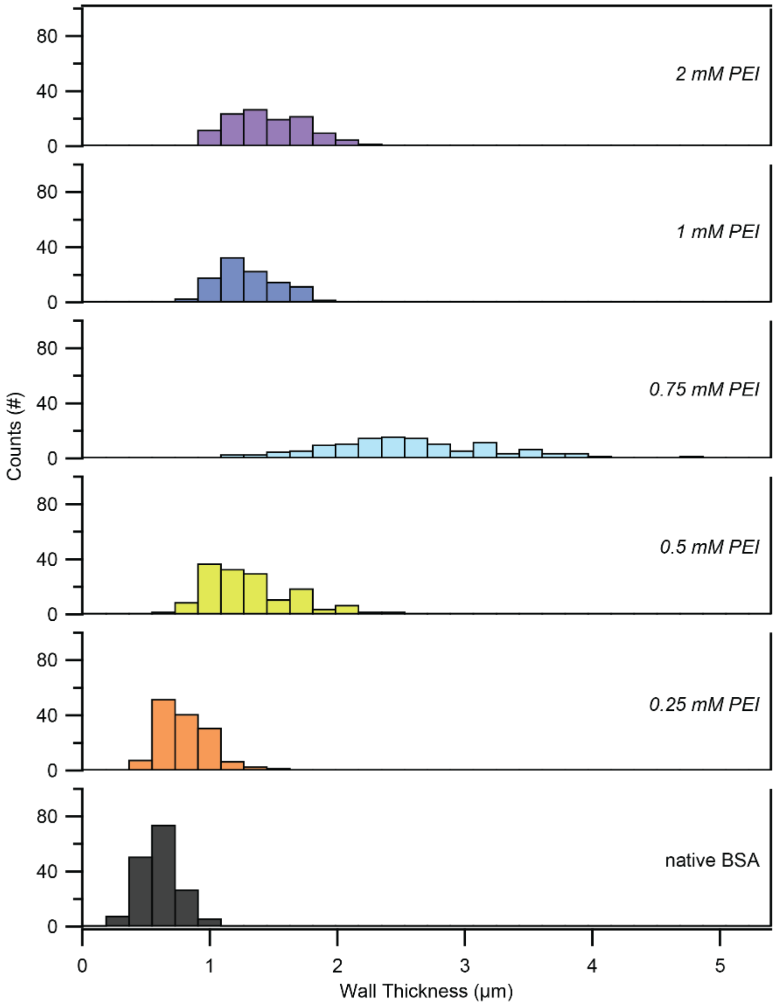

Supplementary Figure 2. Wall-thickness distribution histograms. Wall-thickness distribution histograms of native BSA-based hydrogel and treated BSA-based hydrogel samples with various concentrations of PEI solutions, ranging from 0.25 to 2 mM. The wall thickness was measured from SEM images using ImageJ software.

Validation of BSA (un)folding mechanics inside a BSA-2mM PEI based hydrogel sample

To monitor the (un)folding mechanics of BSA domains inside a BSA-2mM PEI based hydrogel sample, a BSA-based hydrogel was first immersed in TRIS solution with 6.25 μM of 8-anilino-1-naphthalenesulfonate (ANS) for 30 minutes at room temperature (RT). The hydrogel sample fluoresces due to the folded proteins, where ANS produces a FRET signal from excited aromatic amino acids via UV light (Supplementary Figures 3 and 4), measured on G-box instrument (Syngene).

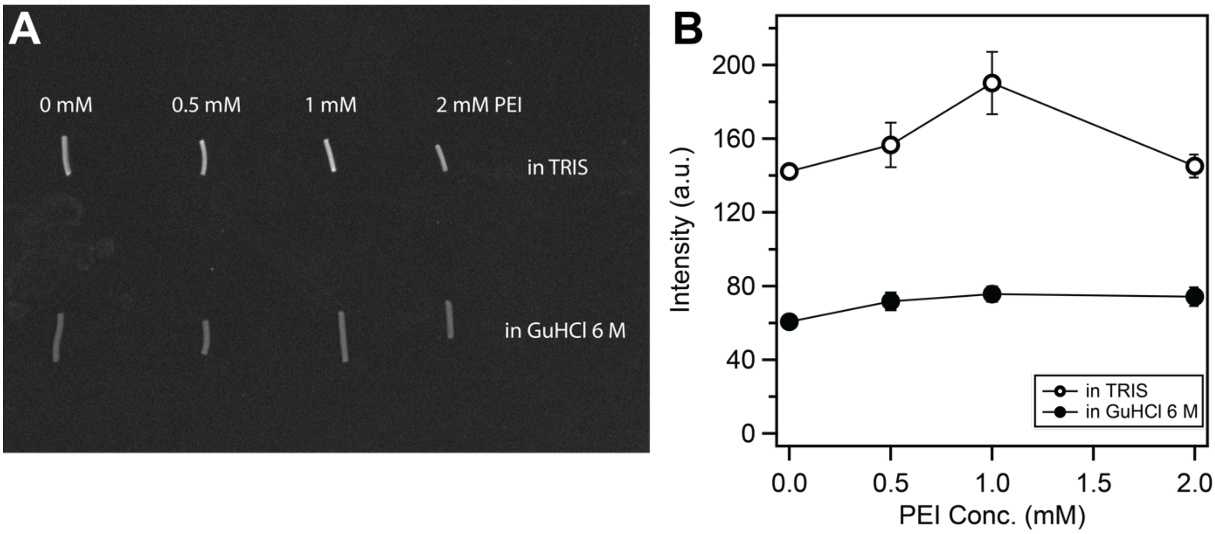

Supplementary Figure 3. BSA protein folding inside BSA-based hydrogels treated with PEI. A) Fluorescence image of BSA hydrogels treated with PEI from 0 to 2 mM (left to right) in native buffer (TRIS – top row) and in denaturing buffer (GuHCl 6 M – bottom row), incubated for 30 min in 6.25 μM ANS. B) Analysis of the gels from (A). PEI concentration does not have a significant effect on the number of unfolded domains. Error bars are S.D. from n = 3 independent hydrogel samples.

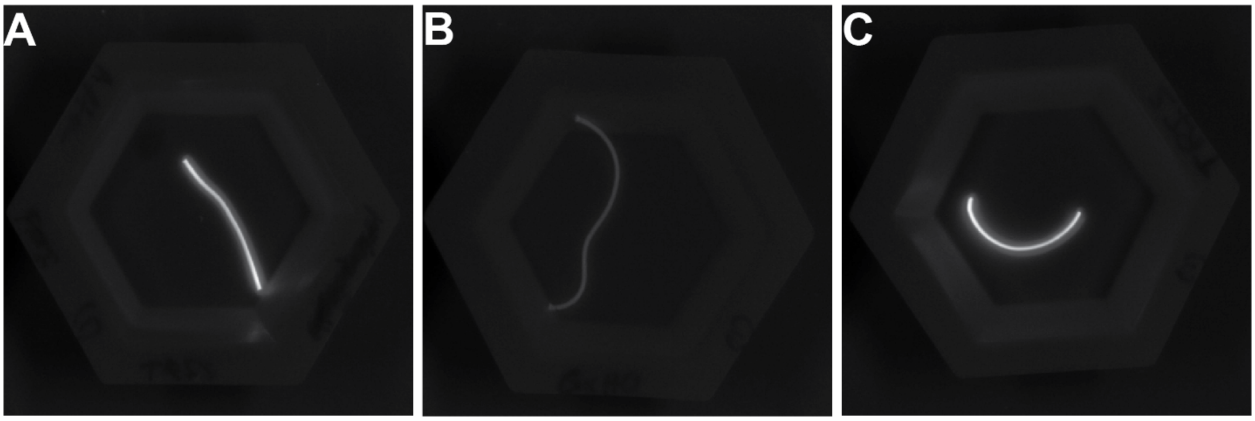

Supplementary Figure 4. Measurement of the folding state of BSA inside hydrogel in various solution conditions. (A) A BSA-2mM PEI based hydrogel sample with ANS immersed in TRIS solution. (B) Same sample from (A) immersed in 6 M GuHCl solution. (C) The hydrogel sample washed from GuHCl salts, them immersed back in TRIS with ANS showing the folding recovery of BSA molecules.

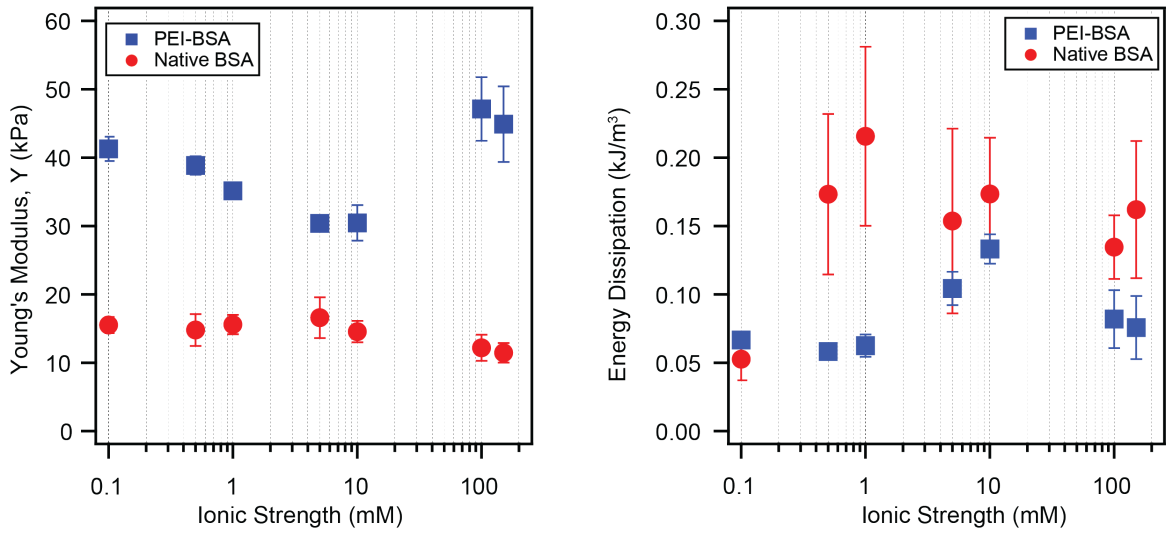

Supplementary Figure 5. Effect of ionic strength on the behavior of BSA hydrogels (native and treated). Measured Young's modulus (A) and energy dissipation (B) of BSA hydrogels treated with 2 mM PEI (blue points) and native (not treated) BSA hydrogels (red points) as a function of ionic strength. Error bars are S.D. from n = 3 independent hydrogel samples.

Supplementary Movie 1. **Movie compilation to explain the shape-memory recovery mechanism of a programmed BSA-based hydrogel treated with 2 mM PEI**. Hydrogel programmed in a spring shape is added into a chemical denaturing GuHCl 6 M solution (at t = 13 s). From t = 27 s time-laps movie (with 1-2 frames-per-second in series of 5 min time intervals) shows the deformation of the spring shape. After 30 min in GuHCl, the hydrogel was moved to TRIS solution using a medical tweezers (t = 1:56 s in the movie). From t = 2:03 s time-laps movie (with 1-2 frames-per-second in series of 5 min time intervals) shows the recovery of the spring shape in regular TRIS buffer, as the BSA domains refold back to their native state. This movie supplements Figure 4.
